# Single-dose application of antithrombin III as alternative anticoagulation during extracorporeal therapy in critically ill patients with advanced liver cirrhosis: a retrospective data analysis

**DOI:** 10.1186/cc9543

**Published:** 2011-03-11

**Authors:** R Brunner, C Madl, W Druml, U Holzinger

**Affiliations:** 1 Medical University of Vienna, Austria

## Introduction

Adequate anticoagulation is essential to achieve efficient and cost-effective renal and liver replacement therapy. However, critically ill patients with advanced liver cirrhosis are associated with low antithrombin III (ATIII) serum levels and increased tendency to both coagulation and bleeding disorder. Thus, we hypothesized that single-dose application of antithrombin III prolongs filter lifetime during renal and liver replacement therapy in critically ill patients with advanced liver cirrhosis without causing additional bleeding problems.

## Methods

In this retrospective study, data of 33 extracorporeal therapies in nine critically ill patients with advanced liver cirrhosis admitted to a medical ICU in 2007 and 2008 were analyzed. Included patients underwent either continuous renal replacement therapy (CRRT), intermittent hemodialysis (IHD) or liver replacement using the molecular adsorbents recirculation system (MARS) with single doses of ATIII as sole anticoagulant. Bleeding complications and filter lifetimes were used as outcome parameters.

## Results

Data were available for 13 CRRT, 14 IHD, and six MARS filters with total filter lifetimes of 661 (CRRT), 66 (IHD), and 42 hours (MARS), respectively. Mean filter lifetimes were 44.0 ± 27.9 (CRRT), 4.7 ± 1.6 (IHD), or 4.6 ± 12.6 hours (MARS). Fifteen percent (two out of 13) of CRRT filters, 7% (one out of 14) of IHD filters and 0% (zero out of six) of MARS filters were lost due to clotting of the dialysis circuit. New onset of bleeding was not observed during IHD, MARS and CRRT.

## Conclusions

Our data suggest that single-dose application of ATIII is effective and safe as alternative anticoagulation in critically ill patients with advanced liver cirrhosis. However, prospective controlled trials are necessary to confirm our findings.

